# Fast-track applications: The potential for direct delivery of proteins and nucleic acids to plant cells for the discovery of gene function

**DOI:** 10.1186/1746-4811-1-12

**Published:** 2005-12-15

**Authors:** Michael R Roberts

**Affiliations:** 1 Department of Biological Sciences, Lancaster Environment Centre, Lancaster University, Bailrigg, Lancaster, LA1 4YQ, UK

## Abstract

In animal systems, several methods exist for the direct delivery of nucleic acids and proteins into cells for functional analysis. Until recently, these methods have not been applied to plant systems. Now, however, several preliminary reports suggest that both nucleic acids and proteins can also be delivered into plant cells by very simple, direct application. This promises to open the way for high-throughput screening for gene function in a range of plant species.

## Introduction

The development of assays that permit high-throughput screening for biological function is an essential goal if we are to fully exploit genome sequence information in plants. Such assays might include over-expression or gene silencing, or the determination of cellular and subcellular localisation of mRNAs and proteins. The majority of techniques that currently exist to perform such assays rely on the production of transgenic plants, or vector-based transient transformation assays. Such methods are necessarily labour intensive and time-consuming, limiting the ability of most researchers to carry out genuine 'functional genomics' projects. However, several recent publications describe systems that permit the direct delivery of nucleic acids and proteins into plant cells in a functional state, providing the potential for rapid functional assays.

## Discussion

### Delivery of macromolecules into animal cells

For many years, researchers using animal cell systems have used synthetic nucleic acids to manipulate gene expression. For example, the use of antisense oligodeoxynucleotides to suppress gene expression was first reported over a quarter of a century ago [1]. Single- and double-stranded DNA and RNA molecules can be introduced into mammalian cells by simple direct application to the culture media, or assisted by various transfection reagents, resulting in antisense or siRNA-mediated suppression of gene expression. A range of different modified nucleic acids that bring different characteristics in terms of stability and binding to target sequences are now used, such as morpholinos, locked nucleic acids, peptide nucleic acids, *etc*. [2]. In many cases these are being developed as potential therapeutic agents [2].

More recently, proteins and other macromolecules have been delivered into cells by linking them to so-called protein transduction domains (PTDs). These are short peptide sequences that when added to the N-terminus of a recombinant protein, or conjugated to other molecules, can carry those molecules directly into cells (reviewed in [3]). The best known are found in the HIV-1 transcriptional activator Tat, and the *Drosophila *transcription factor, Antennapedia [3]. PTDs are generally short, polybasic peptide sequences, and artificial polycationic peptides, such as polyarginine are also effective. Importantly, the uptake of molecules tagged with these peptides does not require specific receptors, endocytosis or active transport. The ability of PTDs to carry molecules across membranes is believed to be the result of the physical characteristics of their interactions with lipid bilayers, suggesting that they should work in any system.

In the past, it has generally been assumed that such delivery systems would not work in plant cells, due to the presence of the cell wall and the difficulty of delivery to multicellular, differentiated tissues. However, work in several laboratories has recently shown that in fact, both proteins and nucleic acids can be efficiently delivered into plant cells in a functional form.

### Delivery of macromolecules into plant cells

**Figure 1:**
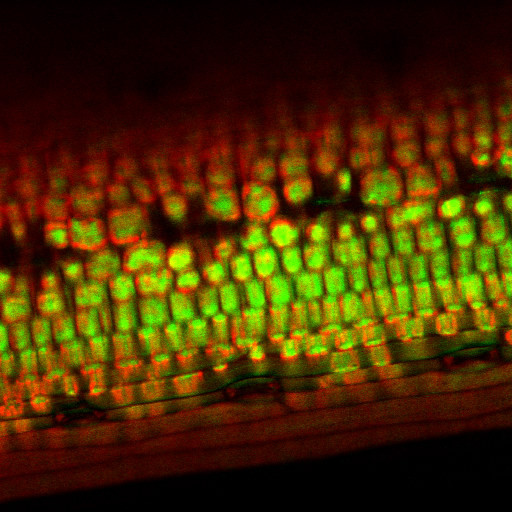
**Oligonucleotides are taken up by intact plant tissues and are distributed throughout the cell**. Confocal microscope image of cells from intact barley leaves following application of an 18-nucleotide oligodeoxynucleotide via the transpiration stream [5]. The oligonucleotide is labelled with the fluorescent dye Alexa Fluor 488 (Molecular Probes), and appears green in the image, whilst chloroplast autofluorescence appears red. Image provided by Professor Christer Jansson, Chuanxin Sun, Anna-Stina Höglund, Helena Olsson and Elke Mangelsen, The Swedish University of Agricultural Sciences.

Unnamalai *et al*. [4], created double stranded RNA (dsRNA) *in vitro*, which was then allowed to complex with a 12mer polyarginine PTD via simple electrostatic interaction. Fluorescent labelling showed uptake of complexes into suspension cultured tobacco cells, characterised by initial accumulation in the nucleus and subsequent redistribution throughout the cytoplasm within 24 h. dsRNAs targeted against the *NPTII *and *GUS *marker genes specifically and substantially reduced gene expression via siRNA-mediated post-transcriptional gene silencing for at least 3 weeks following a 1 h treatment of cells with dsRNA:PTD complexes. An even simpler, but equally effective method for gene silencing was demonstrated by Sun *et al*., [5]. Single-stranded DNA oligonucleotides were taken up by cells of intact barley leaves when fed through the petiole (Figure 1). Fluorescent labelling again showed accumulation first in the nucleus, and then later throughout the cell. An antisense oligonucleotide, but not a complementary sense oligo, silenced gene expression via mRNA degradation [5]. The mechanism involved appears to be hybridisation of the antisense oligo to the mRNA to form an RNA-DNA duplex. RNA-DNA duplexes act as targets for ribonuclease H (RNase H) activity that cleaves RNA around the duplex.

Peptide transduction domains have also been used to deliver proteins into plant cells. Again, the technique employed was remarkably simple and effective. Chang *et al*., [6], produced recombinant GFP proteins in *E. coli*, either alone or tagged with the Tat PTD or a 9mer polyarginine peptide (R9). When these purified proteins were applied to roots of onion or tomato plants, fluorescence rapidly became visible within the nuclei and cytoplasm of cells treated with Tat-GFP and R9-GFP, but not un-tagged GFP. Uptake of PTD-tagged GFP was detectable within 1 min of application, and was maximal in 5 min. Remarkably, cells throughout the root showed fluorescence – not just those in contact with the protein solution. As in animal systems, uptake was not affected by low temperature or inhibitors of endocytosis. GFP fluorescence was maintained for at least 2 days following a 5 min application, suggesting that PTDs are able to deliver proteins that can remain functional for a significant period of time.

## Conclusion

The direct delivery of oligonucleotides and proteins to plant tissues has a range of exciting applications for the discovery of gene function (Table 1). So far, the publications discussed above have included only limited examples of these delivery techniques in plant tissues. An important question that needs to be addressed in the future is whether such molecules can be applied to plants in ways that enables the generation of useful information in a range of biological systems. Clearly the use of suspension cultured cells is limited, and although application through cut petioles may be suitable for short-term molecular and biochemical investigation, it would not permit long-term, developmental studies. Nevertheless, the power of these tools presents an exciting opportunity for further development. They have the potential to enable systematic, high-throughput studies of gene function in a range of plant species.

**Table 1:** Applications of oligonucleotide and protein delivery into intact tissues.

**Molecule**	**Application**	**Reference**
ssDNA	Antisense gene silencing	[2]
dsRNA	Post-transcriptional gene silencing	[7]
Peptide nucleic acids	Inhibition of gene expression by chromosomal interactions	[8]
Proteins	Functional assays	[3]
	Sub-cellular localisation of tagged proteins	

## Competing interests

The author(s) declare that they have no competing interests.
